# Chemical Characterization and Trypanocidal, Leishmanicidal and Cytotoxicity Potential of *Lantana camara* L. (Verbenaceae) Essential Oil

**DOI:** 10.3390/molecules21020209

**Published:** 2016-02-10

**Authors:** Luiz Marivando Barros, Antonia Eliene Duarte, Maria Flaviana Bezerra Morais-Braga, Emily Pansera Waczuk, Celeste Vega, Nadghia Figueiredo Leite, Irwin Rose Alencar de Menezes, Henrique Douglas Melo Coutinho, João Batista Teixeira Rocha, Jean Paul Kamdem

**Affiliations:** 1 Laboratório de Microbiologia e Biologia Molecular, Universidade Regional do Cariri, Crato-CE CEP 63105-000, Brazil; lmarivando@hotmail.com (L.M.B.); duarte105@yahoo.com.br (A.E.D.); flavianamoraisb@yahoo.com.br (M.F.B.M.-B.); mcvegagomez@gmail.com (C.V.); nadghia.fl@gmail.com (N.F.L.); hdmcoutinho@gmail.com (H.D.M.C.); 2 Post-Graduate Program in Biological Sciences, Biochemical Toxicology, Federal University of Santa Maria, Santa Maria-RS CEP 97105-900, Brazil; memypw@yahoo.com.br (E.P.W.); jbtrocha@yahoo.com.br (J.B.T.R.); 3 Program of Post-Graduation in Molecular Bioprospection, Laboratory of Pharmacology and Molecular Chemistry, Chemistry Biology Department, Regional University of Cariri, Crato CEP 63105-000, Brazil; irwin.alencar@urca.br; 4 Departamenro de Bioquímica, Instituto de Ciências Básica da Saúde, Universidade Federal do Rio Grande do Sul, Porto Alegre-RS CEP 90035-003, Brazil

**Keywords:** *Lantana camara*, *Leishmania braziliensis*, *Trypanosoma cruzi*, essential oil

## Abstract

Drug resistance in the treatment of neglected parasitic diseases, such as leishmaniasis and trypanosomiasis, has led to the search and development of alternative drugs from plant origins. In this context, the essential oil extracted by hydro-distillation from *Lantana camara* leaves was tested against *Leishmania braziliensis* and *Trypanosoma cruzi*. The results demonstrated that *L. camara* essential oil inhibited *T. cruzi* and *L. braziliensis* with IC_50_ of 201.94 μg/mL and 72.31 μg/mL, respectively. *L. camara* essential oil was found to be toxic to NCTC929 fibroblasts at 500 μg/mL (IC_50_ = 301.42 μg/mL). The composition of *L. camara* essential oil analyzed by gas chromatography–mass spectrometry (GC/MS) revealed large amounts of (*E*)-caryophyllene (23.75%), biciclogermacrene (15.80%), germacrene D (11.73%), terpinolene (6.1%), and sabinene (5.92%), which might be, at least in part, responsible for its activity. Taken together, our results suggest that *L. camara* essential oil may be an important source of therapeutic agents for the development of alternative drugs against parasitic diseases.

## 1. Introduction

Leishmaniasis is a complex disease known to cause serious public health problems in 88 countries (mainly from Africa, Asia, and Latin America) where the disease was found to be endemic [1]. It is caused by protozoan parasites from more than 21 *Leishmania* species that are transmitted to humans by bites of about 30 species of infected female phlebotomine sandflies [2,3,4].

The complexity of leishmaniasis is probably attributed to its multiple forms, including cutaneous, visceral, and mucocutaneous, which result from the replication of the parasite in macrophages in the mononuclear phagocyte system, dermis, and naso-oropharyngeal mucosa, respectively [5,6]. According to the World Health Organization [1], 12 million of people are affected by the disease and the number of new cutaneous and visceral leishmaniases cases reported are increasing annually. In Brazil, for instance, ten among the fourteen species identified of *Leishmania* have been reported to infect human [7] and about 26,000 new cases of the disease are registered per year [8,9]. This is, at least in part, because the currently-available drugs (e.g., Pentavalent antimony, Paramomycin sulfate) (i) are unaffordable for the developing countries, and (ii) have developed resistance to parasites [6,10,11,12].

Another protozoan disease that is of critical concern in the Latin America is Chagas disease, which is caused by *Trypanosoma cruzi.* Its transmission to human is through feces of infected triatomine insects [13,14]. Like leishmaniasis, the available drugs for the treatment of Chagas disease (nifurtimox and benznidazole) are associated with undesirable effects and are not effective against the chronic forms of the disease [15,16].

Since the last decade, there has been a growing interest from the scientific community for the use of natural therapeutic agents in combating parasitic protozoa diseases including leishmaniasis and trypanosomiasis [17,18,19]. This is because natural therapeutic agents are generally regarded as safe, affordable, and are found to be more effective than synthetic pharmaceuticals in chronic diseases [18,19].

*Lantana camara*, commonly known in Brazil as “camara” and “*camara de espinho*”, is one of the most toxic plants with diverse and broad geographic distribution [20,21,22,23,24]. Its toxicity has been reported in animals [25]. The plant extracts of *L. camara* are used in folk medicine for the treatment of catarrhal infections, cancers, ulcers, asthma, high blood pressure, swellings, tetanus, malaria, chicken pox, bronchitis, respiratory diseases, and rheumatism [21,26,27]. Of pharmacological therapeutic importance, *L. camara* methanolic extract was reported to exhibit anti-leishmanial activity against the promastigote forms of *Leishmania amazonensis* [27]. On the other hand, *L. camara* oil is used for the treatment of skin itches, as an antiseptic for wounds, and externally for leprosy and scabies [22]. In addition, substantial evidence from the literature indicates that essential oil from the leaves of *L. camara* exhibit anti-inflammatory, antibacterial, antifungal, and antimicrobial activities [28,29,30,31].

Although the use of essential oils from plant extracts for the treatment of parasitic protozoa diseases is less investigated, they can be of utmost importance for the development of new drugs against parasitic diseases. Their low density associated with their rapid diffusion across cell membranes (as a result of their liposolubility) can enhance the integration of their active components into the parasites. In this context, the present study aimed to investigate, the trypanocidal, leishmanicidal, and cytotoxic potential of essential oil from the leaves of *L. camara*. Further, chemical characterization of *L. camara* leaf essential oil was performed using gas chromatography-mass spectrometry (GC/MS).

## 2. Results

### 2.1. GC/MS Analysis of L. camara Leaf Essential Oil

Table 1 shows the chemical composition of *L. camara* essential oil analyzed by GC-MS. As it can be seen, twenty seven (27) different compounds representing 98.69% of the total oil were identified. Based on our results, it appears that the major constituents were: (*E*)-caryophyllene (23.75%), bicyclogermacrene (15.80%), germacrene D (11.73%), terpinolene (6.01%), and sabinene (5.92%), while camphene (0.07%), α-terpinene (0.08%), *t*-sabinene-hydrate (0.13%), α-pinene (0.19%), and terpin-4-ol (0.25%) were the less abundant chemicals found in *L. camara* essential oil (Table 1).

**Table 1:** Composition of *Lantana camara* leaf essential oil.

Compounds	RI ^a^	RI ^b^	Oil Composition (%)
α-Pinene	939	937	0.19
Camphene	953	951	0.07
Sabinene	976	675	5.92
β-Pinene	980	983	0.45
Myrcene	991	990	0.31
α-Terpinene	1018	1015	0.08
*p*-Cymene	1026	1026	2.73
(*Z*)-β-Ocimene	1040	1037	0.68
(*E*)-β-Ocimene	1050	1054	0.93
γ-Terpinene	1062	1061	1.84
Terpinolene	1088	1079	6.01
Terpin-4-ol	1177	1174	0.25
α-Terpineol	1189	1193	1.02
*t*-Sabinene hydrate	1254	1257	0.13
α-Copaene	1376	1376	0.93
β-Elemene	1391	1389	1.50
β-Caryophyllene	1404	1401	3.46
(*E*)-Caryophyllene	1418	1423	23.75
Aromandendrene-allo	1461	1460	2.17
α-Humulene	1454	1451	4.04
Germacrene D	1480	1480	11.73
Valencene	1491	1489	8.32
Bicyclogermacrene	1494	1497	15.80
Cubebol	1514	1518	1.47
δ-Cadinene	1513	1509	0.26
Spathulenol	1576	1573	1.98
Caryophyllene oxide	1581	1585	2.67
Total identified (%)	-	-	98.69

Relative proportions of the essential oil constituents were expressed as percentage. ^a^ Retention indices from literature [32]; ^b^ Retention indices experimental (based on homologous series of *n*-alkane C_7_–C_30_).

### 2.2. Effect of L. camara Leaf Essential Oil against T. cruzi

*L. camara* essential oil inhibited *T. cruzi* growth as depicted in Table 2. At the highest concentration tested (250 µg/mL), *L. camara* essential oil reduced the number of the parasites by almost 70%, when compared to control group. Nifurtimox (50 µg/mL), which was used as standard drug against the epimastigotes of *T. cruzi,* killed about 93% of *T. cruzi*. The IC_50_ values (concentration required to kill or inhibit the growth of parasites by 50%) for epimastigotes of *T. cruzi* were 3.02 and 201.94 µg/mL for nifurtimox and *L. camara* essential oil, respectively.

**Table 2:** Activity of essential oil from leaves of *L. camara* against *T. cruzi*.

Nifurtimox (µg/mL)	%AE	Essential Oil (µg/mL)	%AE
-	-	250	67.39 ± 0.26
-	-	125	22.04 ± 5.89
100	100 ± 0.46	-	-
-	-	62.5	0 ± 3.06
50	93 ± 0.66	-	-
10	84 ± 0.62	-	-
1	43 ± 0.93	-	-
0.5	13 ± 2.50	-	-
0.1	0 ± 1.54	-	-
IC_50_ (µg/mL)	3.02 ± 0.75		201.94 ± 1.2

%AE: percentage of epimastigotes of *T. cruzi* killed after treatment with nifurtimox or *L. camara* essential oil. Results are the mean of *n* = 3 independents experiments performed in triplicate.

### 2.3. Effect of Essential Oil from L. camara Leaves against Leishmania braziliensis

Essential oil from the *L. camara* leaves killed the promastigotes of *Leishmania braziliensis* in a concentration-dependent manner (Table 3). Of particular therapeutic importance, 100 µg/mL of *L. camara* essential oil killed 100% of the *L. braziliensis* promastigote forms of the parasite, while the standard drug used (pentamidine) killed 94% of the promastigotes. However, pentamidine was more effective than *L. camara* essential oil, since the concentration needed to kill 50% (IC_50_) of the parasites was 5.69, whereas the IC_50_ for the essential oil of *L. camara* was 72.31 µg/mL.

**Table 3:** Activity of *L. camara* leaf essential oil against *Leishmania braziliensis*.

Pentamidine (µg/mL)	%AP	Essential Oil (µg/mL)	%AE
-	-	250	100 ± 0.76
-	-	125	100 ± 1.25
-	-	100	100 ± 2.23
100	93.9 ± 0.3	-	-
-	-	80	94.95 ± 1.46
-	-	70	36.4 ± 2.22
-	-	62.5	16.44 ± 0.90
-	-	50	15.9 ± 1.50
50	93.9 ± 0.1	-	-
25	89.2 ± 0.6	-	-
12.5	80.6 ± 0.2	-	-
6.25	54.2 ± 0.3	-	-
3.125	15.5 ± 1.1	-	-
IC_50_ (µg/mL)	5.69 ± 0.42		72.31 ± 0.89

%AP: percentage of promastigotes of *L. braziliensis* killed by pentamidine or essential oil of *L. camara.*; %AE: percentage of epimastigotes of *T. cruzi* killed after treatment with pentamidine or *L. camara* essential oil. Results are the mean of *n* = 3 independents experiments performed in triplicate.

### 2.4. Effect of L. camara Leaf Essential Oil on NCTC929 Fibroblasts

The cytotoxic potential of essential oil from *L. camara* in NCTC929 fibroblasts is shown in Table 4. Essential oil of *L. camara* at a concentration of 500 µg/mL completely killed the fibroblasts, while the same effect was observed for nifurtimox (the reference drug) at concentrations ranging from 200 to 600 µg/mL. The order of effectiveness of killing the fibroblast was: nifurtimox (IC_50_ = 82.39 µg/mL) > *L. camara* essential oil (IC_50_ = 301.42 µg/mL) (Table 4).

**Table 4:** Toxicity of Effect of *L. camara* leaf essential oil on NCTC929 fibroblast.

Nifurtimox (µg/mL)	%C	Essential Oil (µg/mL)	%C
600	100 ± 4.4	-	-
-	-	500	100 ± 1.49
400	100 ± 3.8	-	-
-	-	250	14.57 ± 0.72
200	100 ± 0.7	-	-
-	-	125	7.28 ± 1.18
100	64 ± 1.7	-	-
-	-	62.5	6.06 ± 7.72
50	7.0 ± 2.3	-	-
-	-	31.25	0.0 ± 4.09
25	1.4 ± 1.4	-	-
IC_50_ (µg/mL)	82.39 ± 2.16		301.42 ± 3.1

%C: percentage of NCTC929 fibroblasts killed by nifurtimox or essential oil of *L. camara.* Results are the mean of *n* = 3 independents experiments performed in triplicate.

## 3. Discussion

There are an increased interest in finding alternative drugs from the plant kingdom for the treatment of neglected parasitic diseases, in an attempt to replace or supplement those in current use [17,18,33]. Nowadays, phytochemicals are being synthesized and chemically modified to warrant higher potency against these human pathogens [18]. As a pre-requisite for the identification and isolation of active component(s) from plant extracts and/or essential oils, the knowledge of their biological activity is required. In this context, the main objective of the present study was to investigate the biological activities of *L. camara* essential oil with emphasis to its potential to inhibit the promastigote and epimastigote forms of *Leishmania braziliensis* and *Trypanosoma cruzi*, respectively.

Previous studies have reported the leishmanicidal activity of *L. camara* leaf essential oil on promastigote forms of *L. chagasi* and *L. amazonensis* [34] as well as its antibacterial activity [31]. Here, the anti-leishmanicidal activity of *L. camara* essential oil was assessed against the promastigote form of *Leishmania braziliensis.* Comparing our results with that obtained by Machado *et al.* [34], it is possible to extrapolate that *L. camara* essential oil was more effective against *L. amazonensis* (IC_50_ = 0.25 μg/mL) and *L. chagasi* (IC_50_ = 18 μg/mL) than *L. braziliensis* (IC_50_ = 72.31 μg/mL) used in this study. Similar observation was reached by Morais-Teixeira *et al.* [35] when using meglumine antimoniate against the three species of *Leishmania*. In a region of Tunisia (Sned region) endemic to leishmaniasis, Ahmed *et al.* [36] showed that essential oils obtained from *Thymus hirtus* is significantly active against both *Leishmania major* and *L. infantum*, while that of *Ruta chalepensis* was only active against *L. infantum*. The difference in the effectiveness of these oils against different species of *Leishmania* can possibly be attributed to their distinct chemical composition.

The ability of *L. camara* essential oil to inhibit the epimastigote form of *Trypasomia cruzi* was evaluated for the first time. The results demonstrated that *L. camara* essential oil at relatively high concentration (250 µg/mL) exhibited 67.39% inhibition against *T. cruzi.* On the other hand, 500 µg/mL of *L. camara* essential oil was highly toxic to NCTC929 fibroblast. The toxicity of essential oil from *L. camara* was possibly related to its triterpenes (*i.e.*, lantadenes) reported to be present in all parts of the plant [37]. However, compounds other than triterpenes may be involved in *L. camara* essential oil toxicity. In line of this, Martínez-Díaz *et al.* [38] demonstrated recently that (*E*)-caryophyllene, which was the major component of *L. camara* essential oil, exhibits potent antiparasitic effect against *T. cruzi*. This result suggests that (*E*)-caryophyllene might be at least in part, responsible for the observed anti-parasitic activity. However, we cannot rule out the fact that minor and major compounds from *L. camara* essential oil should have made significant contribution to the oil’s activity.

Recently, Charneau *et al.* [39] screened Brazilian Cerrado plant extracts for their anti-protozoan activity. They demonstrated that eight extracts from *Connarus suberosus*, *Blepharocalyx salicifolius*, *Psidium laruotteanum*, and *Myrsine guianensis* exhibited high anti-protozoan activity with IC_50_ values lower than 10 μg/mL. Similarly, Costa *et al.* [40] showed that essential oils obtained from the leaves of species of Annonaceae family, specifically, *Annona pickelli* and *A. salzmannii*, have potent trypanocidal activity against *Trypanosoma cruzi* with IC_50_ value lower than 100 μg/mL. If we compare our results with that obtained by Charneau *et al.* [39] and Costa *et al.* [40] under a similar assay system, we can presume that the anti-*Trypanosoma cruzi* activity of *L. camara* leaf essential oil was relatively low (IC_50_ = 201.94 μg/mL).

## 4. Materials and Methods

### 4.1. Chemicals

Resazurin sodium salt was obtained from Sigma (St. Louis, MO, USA) and stored at 4 °C and protected from light. A solution of resazurin was prepared in 1% phosphate buffer, pH 7, and filter sterilized prior to use. Chlorophenol red-β-d-galactopyranoside (CPRG; Roche, Indianapolis, IN, USA) was dissolved in 0.9% Triton X-100 (pH 7.4). Penicillin G (Ern, S.A., Barcelona, Spain), streptomycin (Reig Jofré S.A., Barcelona, Spain), and dimethylsulfate were also used.

### 4.2. Plant Material and Isolation of Essential Oil

The leaves of *Lantana camara* were collected in Padre Cicero, Crato–Ceara (7°22′S; 39°28′W, 492 m above sea level), Brazil, in June 2012. The plant material was identified and the specimen was deposited in the Herbarium Caririense Dárdano de Andrade–Lima, Regional University of Cariri (URCA), under the number 7518.

The essential oil from the dried leaves of *L. camara* was obtained by hydro-distillation using a Clevenger-type apparatus as described by Guenther [41] with small modifications. At the end of the extraction process, the oil was dried over anhydrous sodium sulfate to remove the aqueous phase, and then stored at 4 °C prior to use.

### 4.3. Gas Chromatography Coupled with Mass Spectrometry (GC/MS) Analysis

The essential oil after preparation was submitted to GC/MS analysis in a Varian 3800 Gas Chromatograph (SHIMADZU, Houston, TX., USA) equipped with a fused silica capillary column (25 m × 0.25 mm i.d., film thickness 0.25 μm) coated with SE-54; carrier gas helium, flow rate 1.0 mL/min and with split mode. The injector temperature and detector temperature were 200 °C and 250 °C, respectively. The column temperature was programmed from 50 °C to 300 °C at 4 °C/min. Individual components were identified by matching their 70 eV mass spectra with those of the spectrometer database using the Wiley l-built library and two other computer libraries’ MS searches using retention indices as a pre-selection routine, as well as by visual comparison of the fragmentation pattern with those reported in the literature [32]. The percentage composition was obtained from electronic integration measurements using flame ionization detection (FID), also set at 250 °C. *n*-Alkanes (C_7_–C_30_) were used as reference points in the calculation of relative retention indices (RIs). The concentration of the identified compounds was computed from the GC peak area without any correction factor. GC analyses were equipped with a flame ionization detector (FID) and a J and W Scientific DB-5 fused silica capillary column (30 m × 0.25 mm × 0.25 μm). Injector and detector temperatures were 250 °C and 290 °C, respectively. Hydrogen was used as carrier gas, flow rate 1.0 mL/min, split mode (1:10).

### 4.4. Cell Lines Used

For *in vitro* studies of *T. cruzi*, the clone CL-B5 was used [42]. Parasites were stably transfected with the *Escherichia coli* β-galactosidase gene (lacZ), provided by Buckner F. at the Instituto Conmemorativo Gorgas (Panama, Brazil). Epimastigotes were grown at 28 °C in liver infusion tryptose broth (Difco, Detroit, MI, USA) with 10% fetal bovine serum (FBS) (Gibco, Carlsbad, CA, USA), penicillin (Ern, S.A., Barcelona, Spain), and streptomycin (Reig Jofré S.A., Barcelona, Spain), as described previously [43], and harvested during the exponential growth phase.

Culture of *L. braziliensis* was obtained from the Instituto de Investigaciones en Ciencias de la Salud, Asunción, Paraguay–IICS. The maintenance of the strain, the form of cultivation, and isolation of shape promastigota were performed following the procedures described by Roldós *et al.* [43]. The inhibition assays of promastigotes was performed using the strain of *L. braziliensis* (MHOM/BR/75/M2903), grown at 22 °C in Schneider’s *Drosophila* medium supplemented with 20% FBS.

For the cytotoxicity assays, the fibroblast cell line NCTC929 grown in Minimal Essential Medium (Sigma) was used. The culture medium was supplemented with heat-inactivated FBS (10%), penicillin G (100 U/mL), and streptomycin (100 µg/mL). Cultures were maintained at 37 °C in humid atmosphere with 5% CO_2_. The viability of these strains was assessed according to Roldós *et al.* [43], through the use of resazurin as a colorimetric method.

### 4.5. Trypanocidal Assay

The essential oil from the leaves of *L. camara* was evaluated against epimastigotes forms of *T. cruzi* using cultures that have not reached the stationary phase [44]. Briefly, epimastigotes were seeded at 1 × 10^5^/mL in 200 µL of liver tryptose broth medium. The plates were then incubated with or without different concentrations of *L. camara* essential oil (250, 125, and 62.5 µg/mL) at 28 °C for 72 h, at which time 50 µL of CPRG solution (200 µM) was added. The plates were incubated at 37 °C for an additional 6 h and were then read at 595 nm. Nifurtimox (100, 50, 10, 1, 0.5, and 0.1 µg/mL) was used as reference standard. The efficacy of the essential oil was estimated by calculating the anti-epimastigotes percentage (AE%) as follow: %AE = [(A_exp_ − A_boil_)/(A_cont_ − A_cult_)] × 100, where, A_exp_ = absorbance of the experimental sample; A_boil_ = Absorbance of the blank sample; A_cont_ = Absorbance of the control; A_cult_ = Absorbance of the culture medium. It should be stressed that the essential oil was solubilized in dimethyl sulfoxide (DMSO) prior to the experiment.

### 4.6. Leishmanicidal Assay

Cultures of promastigotes of *Leishmania braziliensis* were grown in 96-well microplates to a concentration of 10^6^ cells/mL. Different concentrations of *L. camara* essential oil (250, 125, 100, 80, 70, 62.5, and 50 µg/mL) previously dissolved in DMSO, was incubated with the parasite for 72 h at 28 °C. The concentration of DMSO in the wells was not higher than 0.01%. The concentrations of the oil were obtained by serial dilutions. At the end of the incubation period, 20 µL of resazurin (2 mM) was added to the plates and the efficacy of the essential oil or the standard drug was evaluated by direct counting of cells. Each test was performed in triplicate. Pentamidine (100, 50, 25, 12.5, 6.25, and 3.125 µg/mL) was used as standard drugs. The results were expressed in percent inhibition of promastigotes (%AP) and compared with untreated control.

### 4.7. Cytotoxicity Assay

NCTC929 fibroblasts were plated in 96-well microplates at a final concentration of 5 × 10^4^ cells/well. The cells were grown at 37 °C in an atmosphere of 5% CO_2_. After that, the culture medium was removed and *L. camara* essential oil at different concentrations (500, 250, and 125 µg/mL) and a new culture was performed for 24 h. Then, 20 µL of 2 mM resazurin was added to each well. The plates were incubated for 3 h, and the reduction of resazurin was measured using dual absorbance at wavelengths of 490 and 595 nm. The value of the control (blank) was subtracted. Nifurtimox at concentrations of 600, 400, 200, 100, 50, and 25 µg/mL was used as reference.

### 4.8. Statistical Analysis

Results are expressed as mean ± standard error of mean (SEM) of at least three independent experiments performed in triplicate.

## 5. Conclusion

This study demonstrates for the first time the anti-parasitic effect of *L. camara* essential oil against *L. braziliensis* and *T. cruzi*. However, the oil was also toxic to fibroblasts, indicating its potential toxicity to mammalian cells. Consequently, further studies with isolated compounds from *L. camara* essential oil need to be investigated to elucidate the mechanism(s) underlying its anti-parasitic action.
